# Author Correction: Integrated genomic analyses of *de novo* pathways underlying atypical meningiomas

**DOI:** 10.1038/ncomms16215

**Published:** 2018-04-20

**Authors:** Akdes Serin Harmancı, Mark W. Youngblood, Victoria E. Clark, Süleyman Coşkun, Octavian Henegariu, Daniel Duran, E. Zeynep Erson-Omay, Leon D. Kaulen, Tong Ihn Lee, Brian J. Abraham, Matthias Simon, Boris Krischek, Marco Timmer, Roland Goldbrunner, S. Bülent Omay, Jacob Baranoski, Burçin Baran, Geneive Carrión-Grant, Hanwen Bai, Ketu Mishra-Gorur, Johannes Schramm, Jennifer Moliterno, Alexander O. Vortmeyer, Kaya Bilgüvar, Katsuhito Yasuno, Richard A. Young, Murat Günel

Nature Communications 8: Article number: 14433; DOI: 10.1038/ncomms14433 (2017) Published online 02 14 2017; Updated 04 20 2018

In this Article, a subset of the H3K27ac ChIP-seq data (15 benign meningiomas and 2 dura samples (Sample IDs: MN-297, MN-288, MN-292, MN-163, MN-1037, MN-105, MN-201, MN-249, MN-191, MN-1066, MN-169, MN-291, MN-24, MN-79, MN-1044, CONTROL1, CONTROL2) was reported previously in a publication by the corresponding author^1^. These data were created by Dr. Justin Cotney in Dr. James Noonan’s laboratory at Yale. The GEO database entry associated with this dataset has been updated to reflect this fact (GSE91372).

1. Clark *et al*., Genomic analysis of non-NF2 meningiomas reveals mutations in TRAF7, KLF4, AKT1, and SMO. *Science* **1**, 1077–1080 (2013).

