# Supplementary material for: Integrated genomic analyses of de novo pathways underlying atypical meningiomas
Source: Nat Commun. 2017 Feb 14;8:14433. doi: 10.1038/ncomms14433 (PMC5316884; doi:10.1038/ncomms14433)
Supplement: Supplementary Information — Supplementary Figures and Supplementary References [file ncomms14433-s1.pdf]

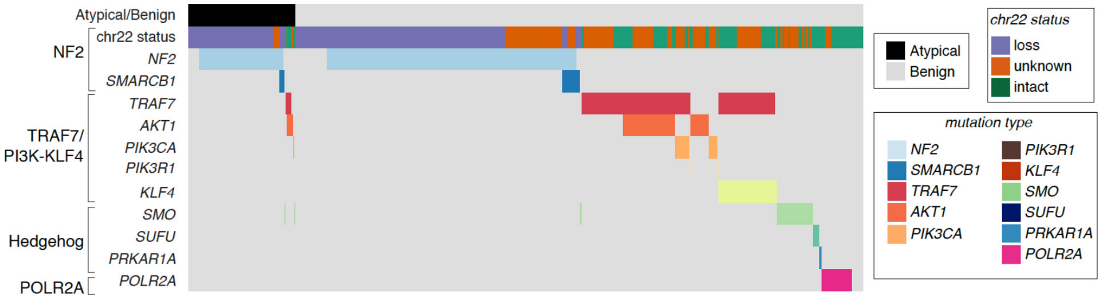

Supplementary Figure 1. Oncoprint depicting the mutational profile of whole exome or targeted sequenced 556 meningiomas. Histological grade, chromosome 22 loss status and recurrently mutated genes are summarized. Symbols for the recurrently mutated genes, which are grouped based on meningioma subgroups, are shown on the left. The color codes are explained on the right.

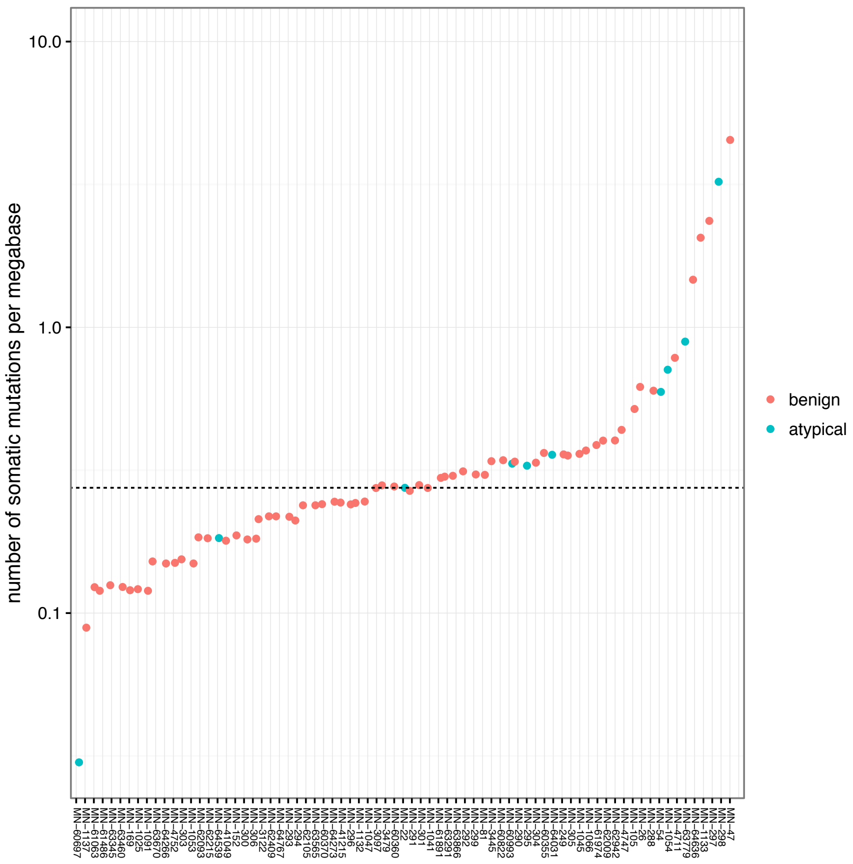

Supplementary Figure 2. Mutation frequency of meningiomas. Every dot represents a sample while the dashed horizontal lines are the median numbers of mutations (salmon:benign,n=65 blue:atypical n=10). The median mutation frequency (median: 0.27) of meningioma is lower than glioblastoma[1].

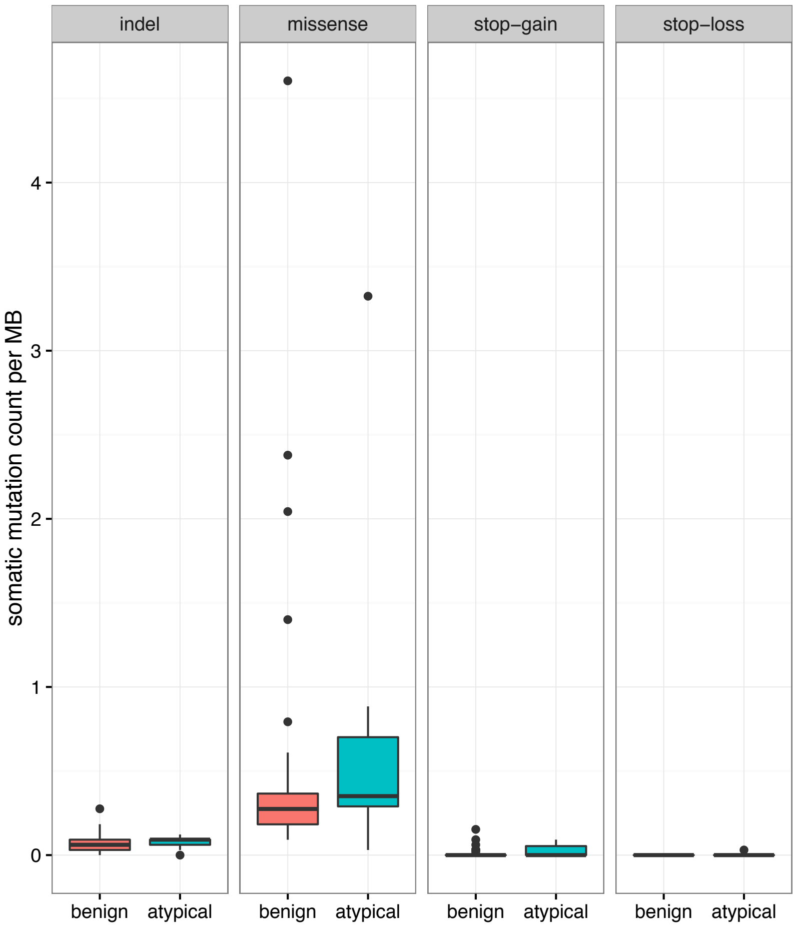

Supplementary Figure 3. Number of somatic indel, missense, stop-gain and stop-count mutation count per MB across atypical and benign meningiomas. Boxplots of the number of somatic indel, missense, stop-gain, stop-loss mutation count from 75 meningioma exomes normalized per millibase of sequencing (n=10 *atypical*, salmon; n= 65 benign, blue). The number of indel, missense, stop-gain and stop-loss somatic mutations from whole-exome sequencing data is not statistically different between *atypical* and other meningioma samples (P=0.71 indel; P=0.37 missense, P=0.18 stop-gain, P=0.34 stop-loss, Student's t-test). Lines depict the median values, boxes plot 25th to 75th percentiles, whereas separately plotted points show the outliers.

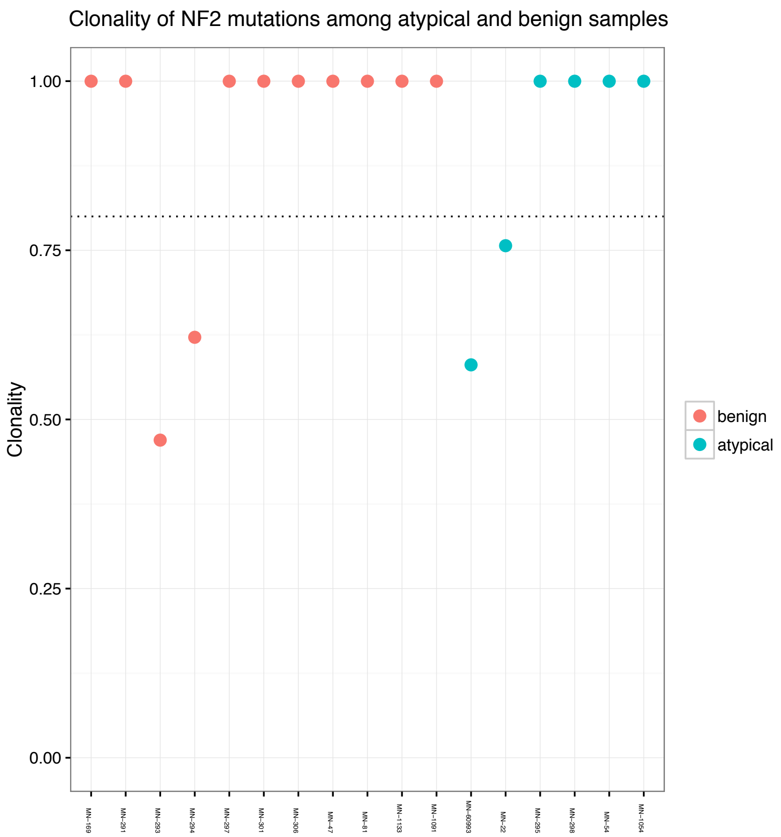

Supplementary Figure 4. **Clonality of *NF2* mutant atypical and benign tumors.** In the majority of our samples, we calculated *NF2* mutations to have a clonality rate of 100%, suggesting that *NF2* mutations indeed occurred early during tumor formation. The sample with the lowest clonality rate was 46%.

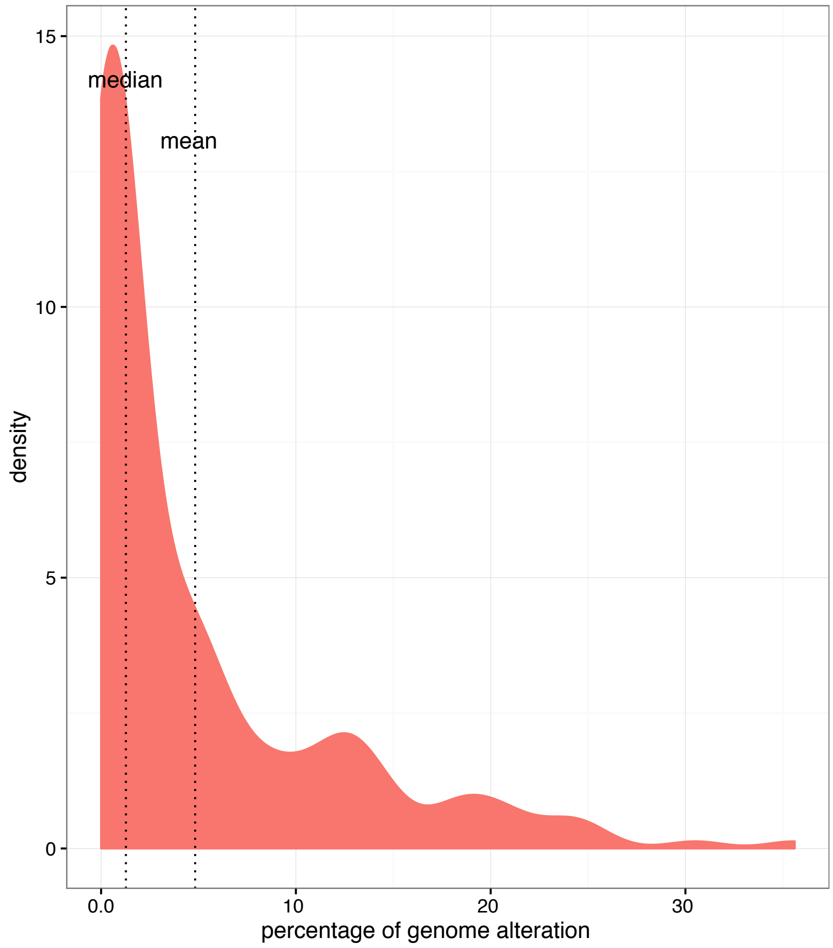

Supplementary Figure 5. The distribution of percentage of genome alteration (PGA) across all (n=208) meningioma samples. The mean (4.82%) and median (1.27%) PGA values are marked as dash lines in the plot.

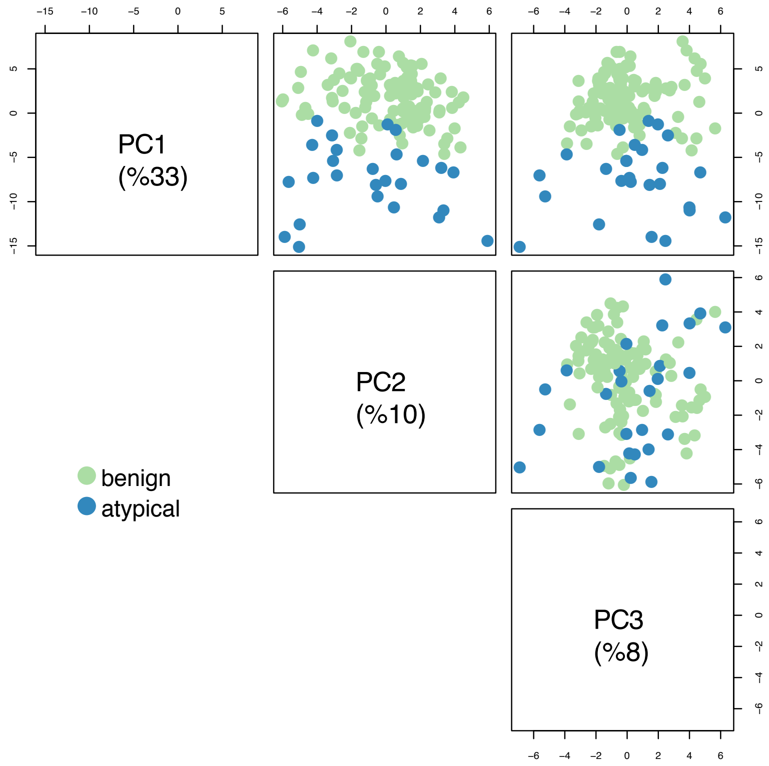

Supplementary Figure 6. Principal component (PC) analysis of mRNA gene expression data. Principal component (PC) analysis of meningioma gene expression data using mRNA signature genes separates atypical and benign samples (n=138).

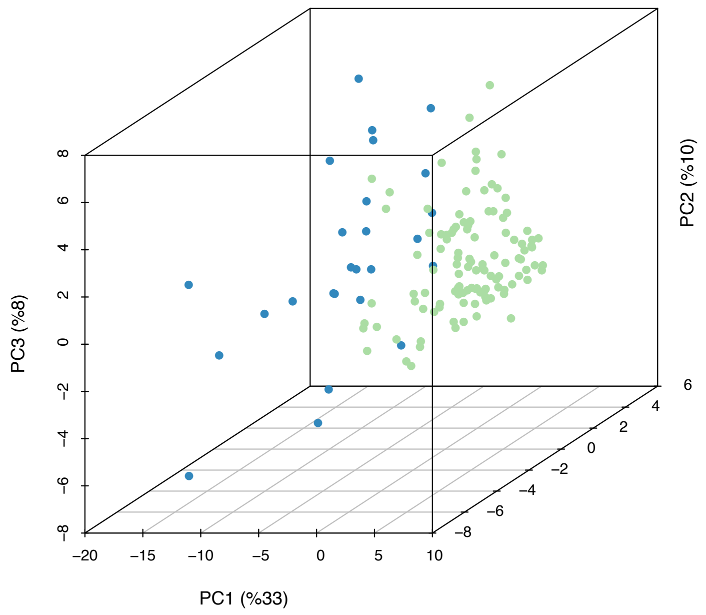

Supplementary Figure 7. Principal component (PC) analysis of mRNA gene expression data. Principal component (PC) analysis of meningioma gene expression data using mRNA signature genes separates atypical and benign samples (n=138).

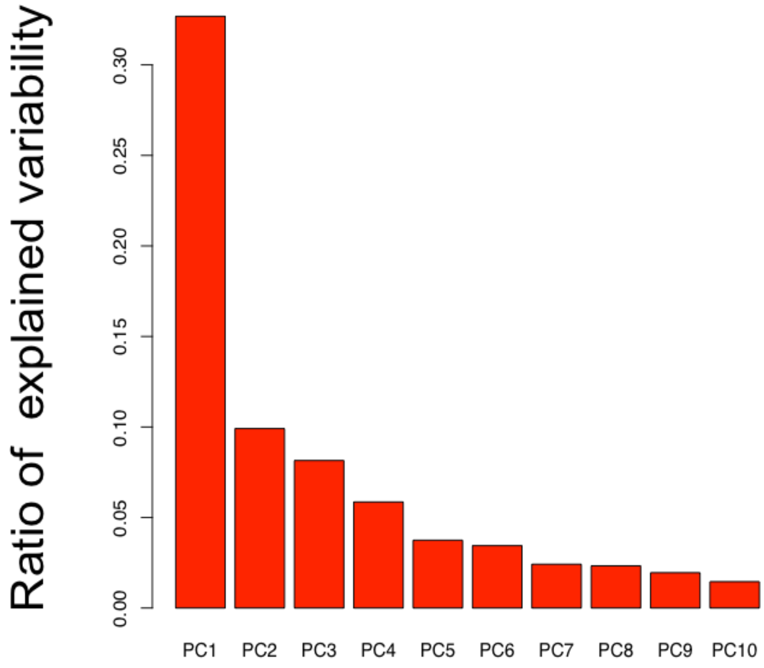

Supplementary Figure 8. Explained percentage of variability. Barplots of the percentage of variability explained by the top 10 principal components.

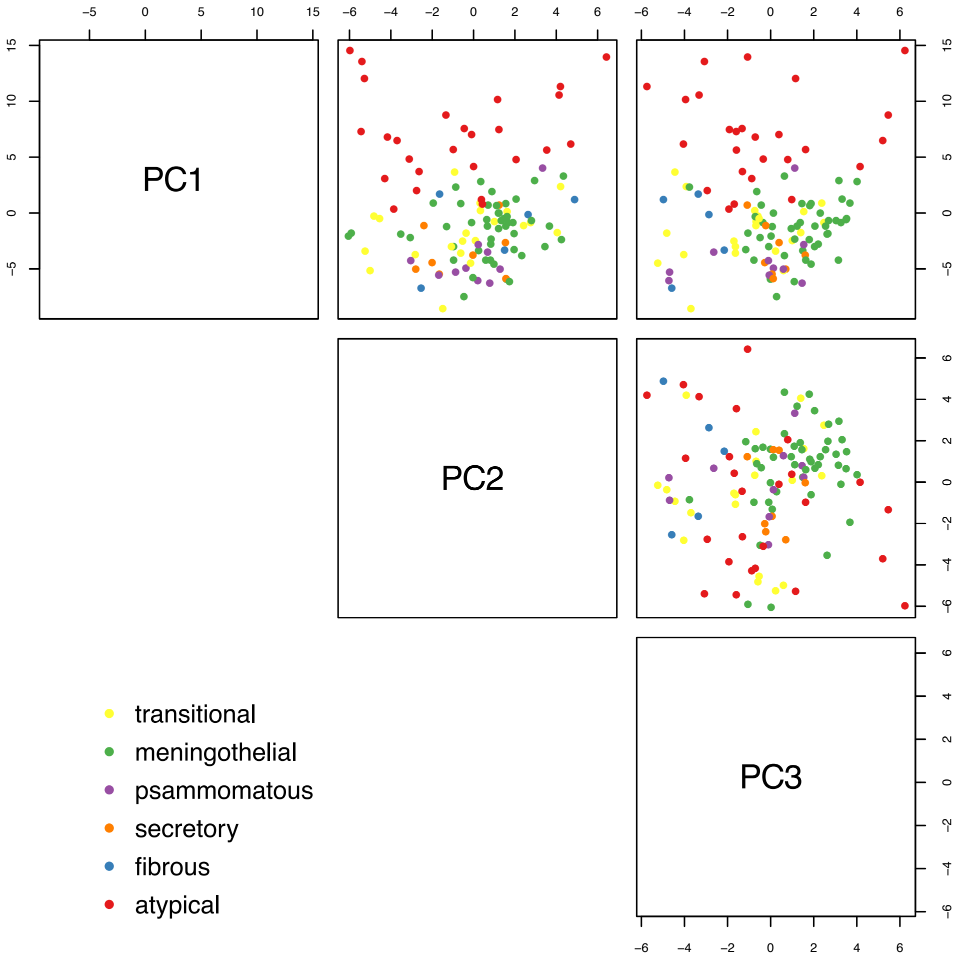

Supplementary Figure 9. Principal component (PC) analysis of gene expression data. Gene expression PCA plot using the mRNA signature genes distinguishes benign *NF2* samples from atypical ones, independent of histological subtype (n=138). Different histology types are color coded, which is shown at the bottom.

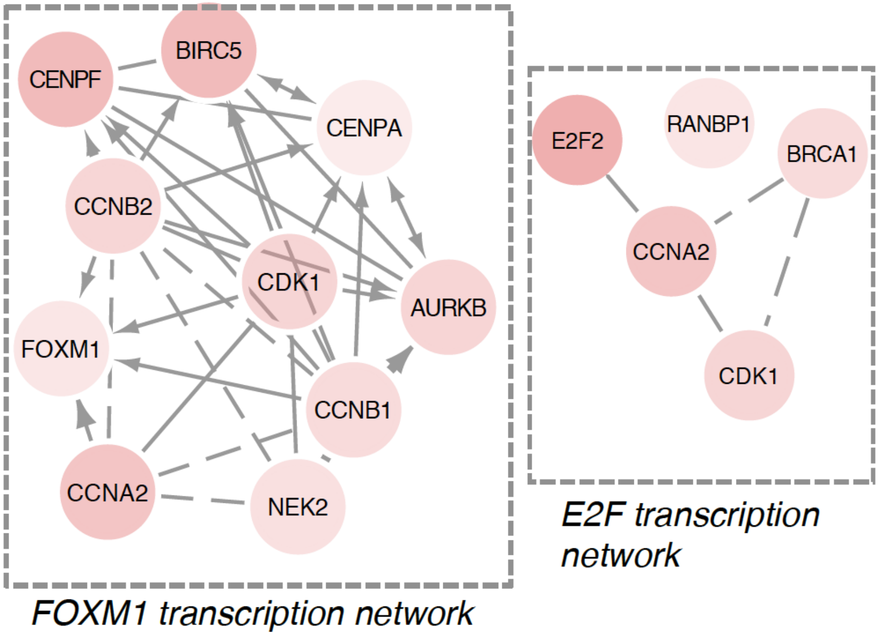

Supplementary Figure 10. GO Enrichment analysis. Significantly enriched pathways based on differential gene expression in atypical meningiomas as compared to benign tumors, which are identified using Cytoscape Reactome plugin, are plotted (n=138).

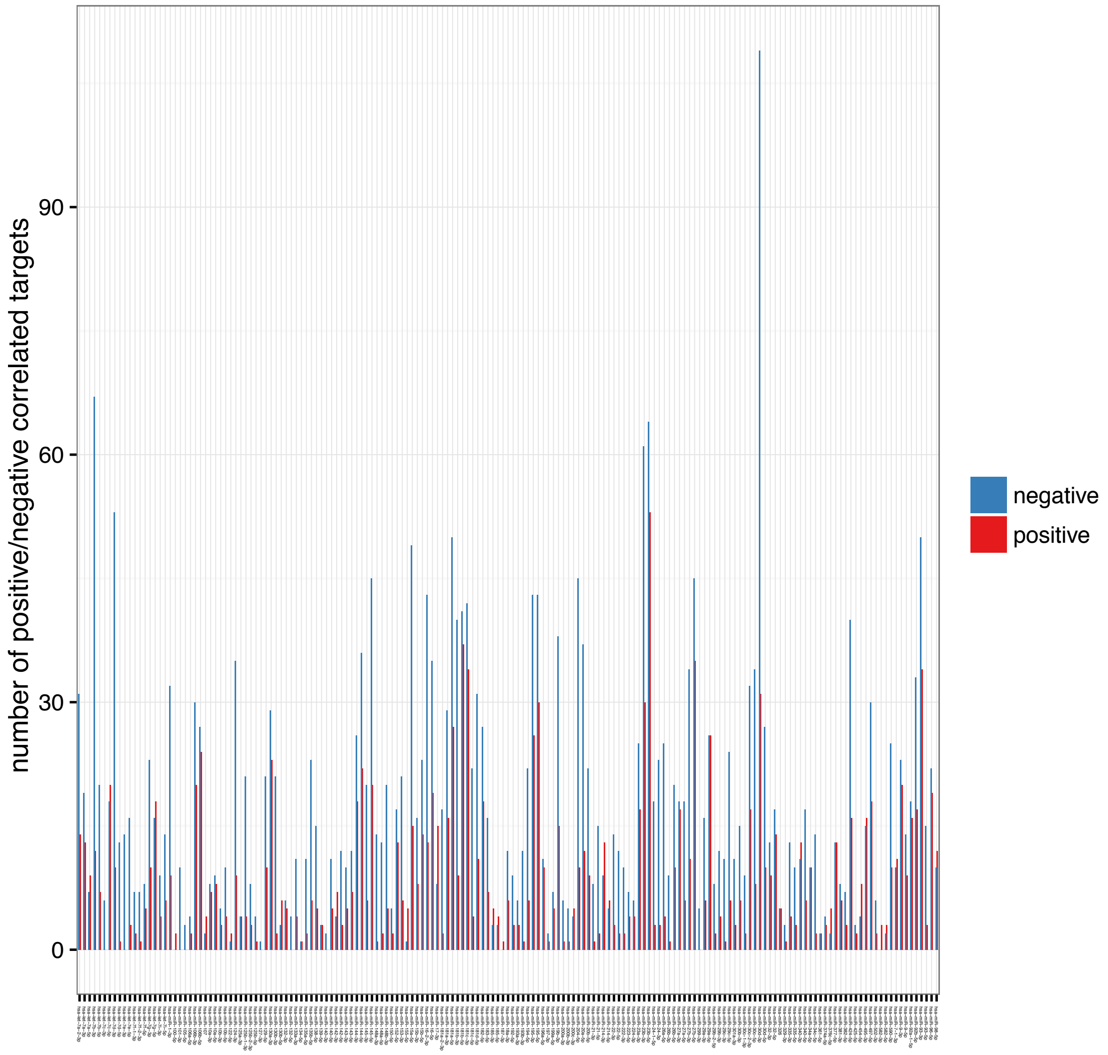

Supplementary Figure 11. Barplots of the number of negatively and positively correlated targets of miRNAs. The number of negatively and positively correlated targets of miRNAs is statistically different (P < 2.2e-16) (negative:blue, positive:red) (n=22). Wilcoxon paired test was used to calculate significance.

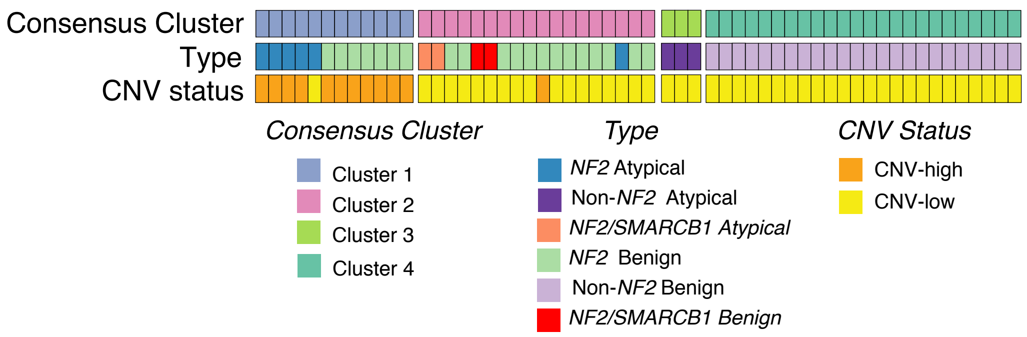

Supplementary Figure 12. DNA Methylation clustering after removing regions affected by CNV events. DNA methylation clustering analysis was not affected by the large-scale chromosomal events; removing the regions that were affected by large scale chromosomal events (chr1, chr14, chr22) , did not change the results of the clustering analysis (n=57 samples).

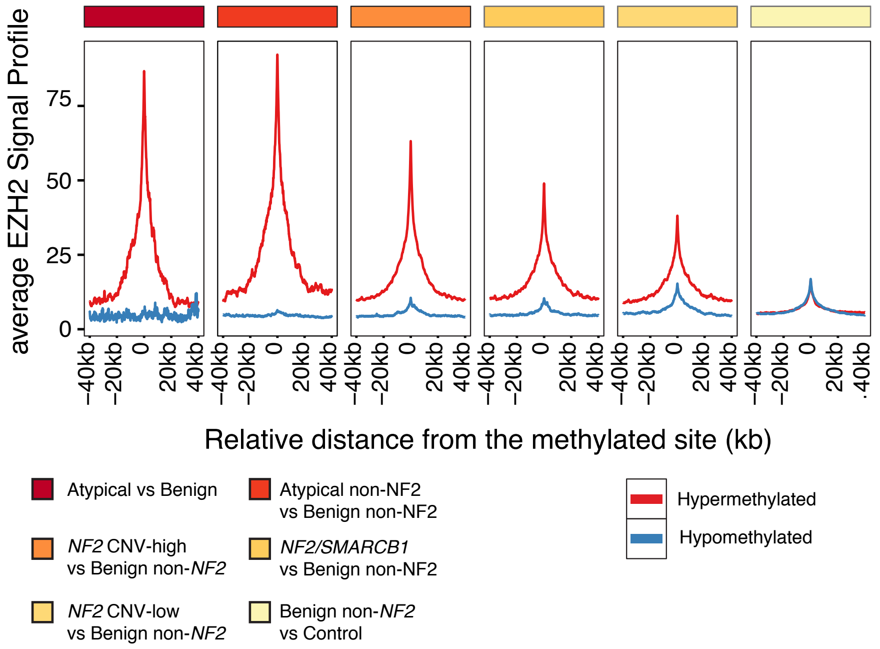

Supplementary Figure 13. Aggregation plot of EZH2 ChIP-seq signals signals. Aggregation plot of EZH2 ChIP-seq signals (previously reported in hESCs[2]) centered at hyper- (red line) and hypo-methylated (blue line) sites are shown. The analyses reveal an increase in hypermethylation of H3K27me3 and EZH2 targets in ESCs in atypical versus benign samples. The level of hypermethylation correlated with the likelihood of being atypical.

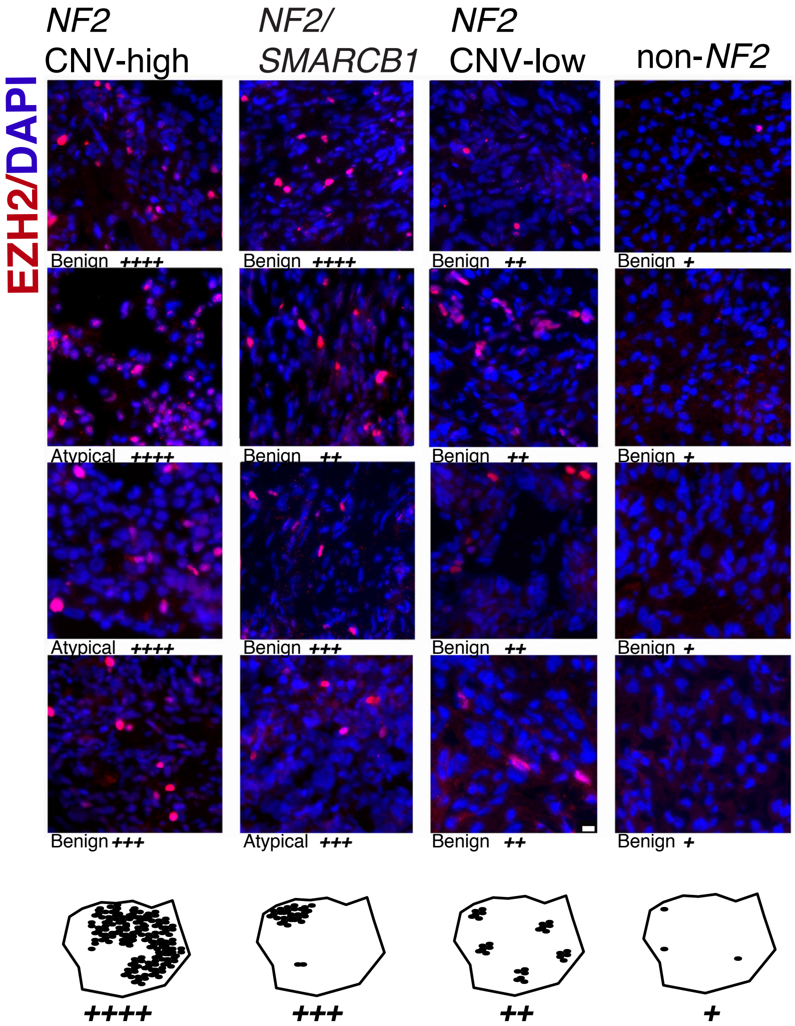

Supplementary Figure 14. Immunofluorescence staining for *EZH2*. Immunofluorescence staining for EZH2 demonstrates increased expression in *NF2* mutant versus non-*NF2* meningiomas. Tumor samples were stained for EZH2 (scale bar is 10um). Expression of EZH2 is depicted in red and DAPI, which marks the nuclei, is shown in blue. EZH2 staining quantifications are summarized using diagrams that are shown below the immunofluorescence staining images.

Supplementary references

1 Alexandrov, L. B. *et al*. Signatures of mutational processes in human cancer. *Nature* **500**, 415-421, doi:10.1038/nature12477 (2013).

2 Consortium, E. P. An integrated encyclopedia of DNA elements in the human genome. *Nature* **489**, 57-74, doi:10.1038/nature11247 (2012).
